# Vulvar Pyogenic Granuloma in a Postmenopausal Woman: Case Report and Review of the Literature

**DOI:** 10.1155/2011/201901

**Published:** 2011-09-08

**Authors:** Deniz Cemgil Arikan, Gurkan Kiran, Hamide Sayar, Bulent Kostu, Ayhan Coskun, Hakan Kiran

**Affiliations:** ^1^Department of Obstetrics and Gynecology, Kahramanmaras Sutcuimam University Medical Faculty, 46050 Kahramanmaras, Turkey; ^2^Department of Pathology, Kahramanmaras Sutcuimam University Medical Faculty, 46050 Kahramanmaras, Turkey; ^3^Department of Obstetrics and Gynecology, Sereflikochisar State Hospital, 06950 Sereflikochisar, Ankara, Turkey

## Abstract

*Introduction*. Although pyogenic granulomas (PG) are common and benign vascular proliferations of the skin and mucous membranes, they are relatively rare on the vulva. *Case Presentation*. A 57-year-old G7P7 postmenopausal woman presented with a 3-year history of a foul smell and bleeding lesions in the genital region. A gynecologic examination revealed multiple large papillomatous, pedunculated, and lobulated lesions that were cherry-red and infective in appearance. There was a 2-cm lesion at the upper intersection of the labia majora, a 2-cm lesion on the right labium majus, and a 4-cm lesion on the clitoris. The patient complained of itching, and the lesions were asymptomatic, except for occasional bleeding. All lesions were excised and sent for histopathological examination, which revealed an ulcerated polypoidal structure with extensive proliferation of vascular channels lined by a single layer of endothelium. The histopathological features were consistent with PG. *Conclusion*. The present case is the first case of multiple pyogenic granulomas on the vulva in a postmenopausal woman.

## 1. Introduction

Pyogenic granulomas (PGs) (lobular capillary hemangioma) are common and benign vascular proliferations of the skin and mucous membranes [1]. Clinically, PG is a sessile or pedunculated, single erythematous, friable polypoid, and exophytic lesion, with a smooth or lobulated surface, that bleeds easily after minor trauma [2, 3]. The lesions are mostly painless, or they can be slightly tender [3]. The lesions are frequently found in the oral mucosa or on the trunk or limbs [3]. 

Pyogenic granulomas of the vulva are a relatively rare finding, and a limited number of cases—to our knowledge, there are two reported cases—have been reported in the literature [4, 5]. In this case report, we present for the first time a woman in the postmenopausal period with multiple PGs of the vulva. 

## 2. Case Report

**Figure 1:**
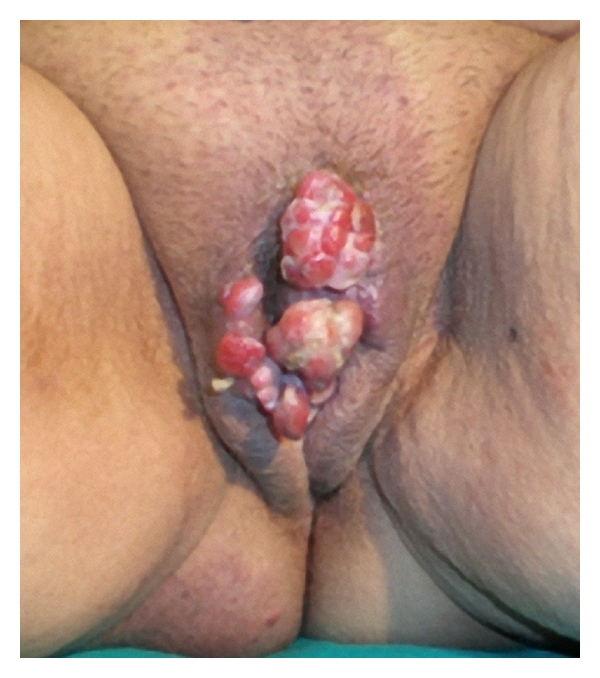
Appearance of multiple pyogenic granulomas of the vulva.

A 57-year-old G7P7 postmenopausal woman presented with a 3-year history of a foul smell and bleeding lesions in the genital region. A gynecologic examination revealed multiple large papillomatous, pedunculated, and lobulated lesions that were cherry-red and infective in appearance. There was a 2-cm lesion at the upper intersection of the labia majora, a 2-cm lesion on the right labium majus, and a 4-cm lesion on the clitoris (Figure 1). There was no vaginal or cervical pathology. An ultrasound examination revealed a normal uterus and adnexa. The patient complained of itching, and the lesions were asymptomatic, except for occasional bleeding. There was no history of any trauma other than that caused by itching for 2-3 years. The tissue smear and Gram smear from the lesions did not reveal any organisms. Serology for herpes, syphilis, and human immune deficiency viruses were negative. There was no palpable inguinal or pelvic lenfadenopathy.

**Figure 2:**
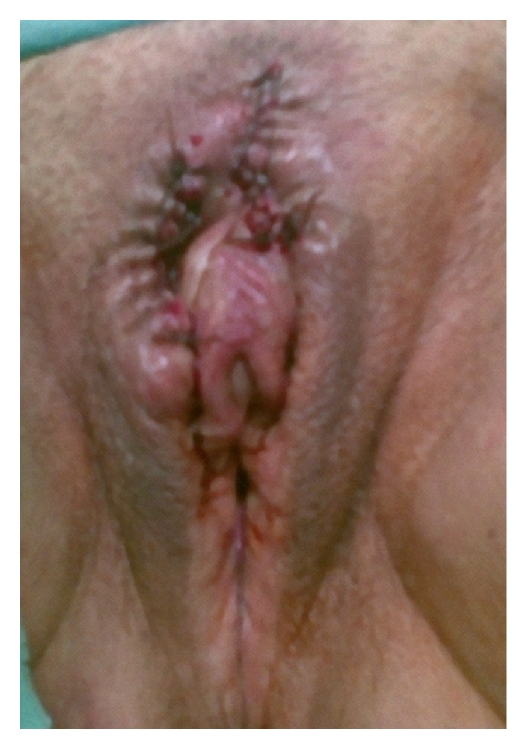
Postoperative view of the vulva.

**Figure 3:**
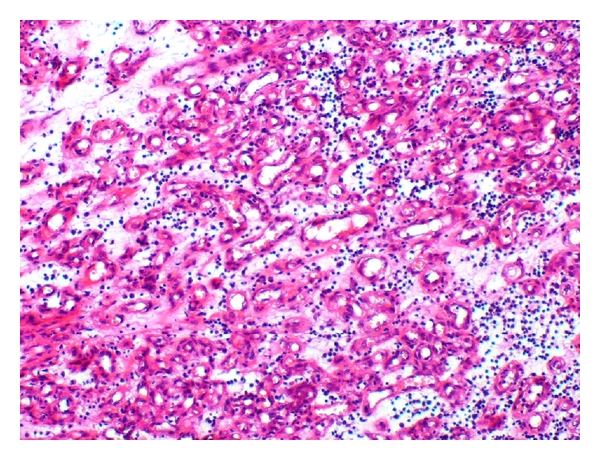
The vascular channels lined by a single layer of endothelium surrounded with lymphomononuclear cells (hematoxylin and eosin stain, original magnification ×100).

All lesions were excised and sent for histopathological examination (Figure 2), which revealed an ulcerated polypoidal structure with extensive proliferation of vascular channels lined by a single layer of endothelium (Figure 3). The intervening stroma, consisting of collagen, was infiltrated by lymphomononuclear cells. The histopathological features were consistent with PG. There was no noticeable recurrence until six months after the excision.

## 3. Discussion

Although it is believed that PG is a reactional benign lesion formed due to minor trauma and/or chronic low-grade local irritation [6–8], its etiology is still unclear. Recently, it has been considered to be a reactive hyperproliferative vascular response to a variety of stimuli, rather than a neoplastic or infectious process [9]. Minor traumas or underlying cutaneous diseases could cause an excessive local production of angiogenic growth factors or cytokines, which could be an important factor in the pathogenesis of PG [10, 11]. It has been reported that minor trauma related to zipper accidents, sexual intercourse, and circumcision has played an important role in the etiology of PG of the male genitalia [9]. In a case presented by Gupta et al. [4], although they did not definitely know whether the pruritic disease itself or excoriation trauma was the stimulus for the angiogenesis, they thought that the disease had precipitated the PG. In our case, a history of itching due to atrophy of the vulva as a result of menopause could cause chronic irritation and, as a result, hasten the growth of lesions (Table 1). 

Although PGs are seen in every age group, a higher frequency of PG is observed in the second decade of life [6], with a female predilection of 2 : 1 [12], probably because of the vascular effects of female hormones (high estrogen and progesterone) [7]. There is a study in which estrogen and progesterone receptor positivity has been demonstrated in mucosal PGs [13]. However, in a case of cutaneous PG during pregnancy reported by Rumelt et al. [14] and in a study of 21 cutaneous PGs reported by Nichols et al. [15], estrogen and progesterone receptors were found to be negative. In another study, the distribution of cutaneous PGs were found to be equal in both sexes, and the researchers concluded that estrogen had no effect on the development of cutaneous PGs [16]. As seen from the reports, cutaneous PGs are not associated with hormonal status [14–16]. As our case was the first vulvar (cutaneous) case in the postmenopausal period, we think it supports the hypothesis that estrogen does not play a role in the etiology of cutaneous PGs.

Although PG usually occurs as a single lesion [4], there were multiple lesions in the present case. Pyogenic granulomas of the vulva reported in the literature are all multiple [4, 5]. Although multiple eruptive PGs can occur after excision or in the presence of underlying cutaneous pathology [16, 17], there were no predisposing factors in our case or in other cases of multiple vulvar lesions presented in the literature [4, 5]. Pyogenic granuloma lesions may be seen in any size from a few millimeters to a few centimeters [3]. Most are small, less than 5 mm in diameter, and grow rapidly over several weeks [18]; however, the vulvar lesions in our case and in the other two cases were large lesions [4, 5] (Table 1). 

## Figures and Tables

**Table 1:** Reports of pyogenic granuloma of vulva.

Cases	Age	Trauma?	Number of lesions (multiple or single)	Location	Symptom	Treatment	Recurrence
Gupta et al. [4]	21	Itching	Multiple	Labia majora	Bleed	Total excision	No
Kaur et al. [5]	7	No	Multiple	Labia majora, minora, and introitus	Bleed	Total excision	No
Our case	57	Itching	Multiple	Labia majora and clitoris	Bleed	Total excision	No
